# RESTful API for iPTMnet: a resource for protein post-translational modification network discovery

**DOI:** 10.1093/database/baz157

**Published:** 2020-05-12

**Authors:** Sachin Gavali, Julie Cowart, Chuming Chen, Karen E Ross, Cecilia Arighi, Cathy H Wu

**Affiliations:** 1 Center for Bioinformatics and Computational Biology, 205 Delaware Biotechnology Institute, 15 Innovation Way, Newark, DE 19711, USA; 2 Department of Biochemistry and Molecular & Cellular Biology, 337 Basic Science Building, 3900 Reservoir Road, N.W, Washington D.C. 20057, USA; 3 Department of Computer and Information Sciences, 101 Smith Hall, 18 Amstel Ave Newark, DE 19716, USA

**Keywords:** iPTMnet, RESTful API, Web Service, Rust, Docker, Post-Translational Modification, Phosphoproteomics, Cloud

## Abstract

iPTMnet is a bioinformatics resource that integrates protein post-translational modification (PTM) data from text mining and curated databases and ontologies to aid in knowledge discovery and scientific study. The current iPTMnet website can be used for querying and browsing rich PTM information but does not support automated iPTMnet data integration with other tools. Hence, we have developed a RESTful API utilizing the latest developments in cloud technologies to facilitate the integration of iPTMnet into existing tools and pipelines. We have packaged iPTMnet API software in Docker containers and published it on DockerHub for easy redistribution. We have also developed Python and R packages that allow users to integrate iPTMnet for scientific discovery, as demonstrated in a use case that connects PTM sites to kinase signaling pathways.

## Introduction

iPTMnet (1, 2) is a resource for protein post-translational modification (PTM) knowledge discovery that integrates information extracted from PTM text mining tools such as eFIP (3) and RLIMS-P (4), as well as curated databases (5–17) and ontologies (18). The current iPTMnet release v5.1 consists of more than 63 000 post-translationally modified proteins, 700 000 PTM sites and 1000 PTM enzymes for 11 PTM types, along with 23 000 enzyme-substrate-site triples and 3000 PTM-dependent protein–protein interactions (PPIs) from human and major species. The iPTMnet website (https://research.bioinformatics.udel.edu/iptmnet/) provides features including searching and browsing PTM data, batch retrieval of enzymes and PPIs for the given PTM sites, an integrated sequence alignment viewer and a Cytoscape network view (19). It enables a systems-level analysis of protein PTM networks and conservation across species to facilitate hypothesis generation.

To integrate iPTMnet data into analysis pipelines, there is a need to access the data programmatically. Currently, iPTMnet provides data access only by website navigation. Hence, users need to copy and paste data from the website for integration into their own studies. This manual process is error-prone and time-consuming. An alternative is to provide a bulk download of the underlying database to the interested users. But this would involve learning the iPTMnet database schema and then writing scripts or tools to extract the data from the database. Also, this approach is not scalable as the users will have to modify their scripts every time the database schema changes.

Hence, we have developed the RESTful API to encapsulate and standardize access to the iPTMnet database. The API will streamline the integration of iPTMnet into existing tools and pipelines. It provides well-defined functions to retrieve data for every view on the iPTMnet website. Additionally, we have developed Python and R packages that hide the technical details of using the API to make it easy for biologists to use.

## Materials and Methods

### The architecture of the iPTMnet service

**Figure 1:**
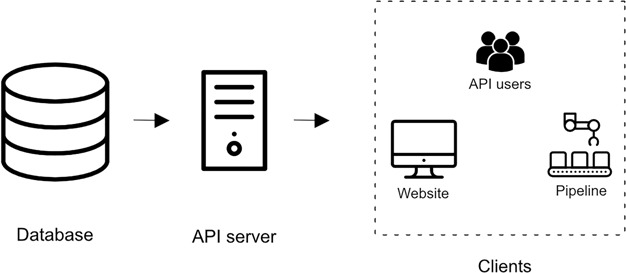
Overview of iPTMnet Architecture. The overall iPTMnet architecture has three distinct layers. The database layer is built using Oracle or Postgres and holds the iPTMnet data. The API server layer is the actual API server software that encapsulates the logic to interpret requests and return a response in an appropriate format. The client layer consists of various frontends like the iPTMnet website or the Python and R packages.

The architecture of the iPTMnet service consists of three different layers: the database layer, the API server layer and the client layer (Figure 1). All three layers of the service are packaged in separate Docker containers (https://hub.docker.com/u/udelcbcb). This allows the development, distribution and deployment of the components in a modular manner.

**Table 1:** Endpoints provided by the iPTMnet API

Endpoints	Description/Summary
/search	Search for proteins in the iPTMnet database
/{id}/info	Retrieve top level info about an iPTMnet entry
/{id}/substrate/	Retrieve the list of PTM sites for the given substrate
/{id}/msa/	Retrieve the annotated multiple sequence alignment for the iPTMnet entry
/{id}/proteoforms/	Retrieve the list of PTM-dependent PPI for the iPTMnet entry
/{id}/ptmppi/	Retrieve the list of PPIs for the proteoforms of the iPTMnet entry
/batch_ptm_enzymes	Retrieve PTM enzyme information for a given list of PTM sites
/batch_ptm_ppi	Retrieve PTM dependent PPI information for a given list of PTM sites
/variants	Retrieve the list of PTM variants for the iPTMnet entry

The database is the foundational layer, upon which all the other layers are built. Our in-house deployment of iPTMnet is backed by Oracle database version 12.0c (https://www.oracle.com/database/index.html). One of our goals in building the iPTMnet API was to support users who want to have the iPTMnet installed locally or on their own private cloud. Hence, we packaged the API server software in a Docker container so that it can be deployed on any local or cloud-based server. To support users who need a free and open-source database, we have built the iPTMnet API to work with PostgreSQL (http://www.postgresql.org). The database engine can be configured at runtime by changing the ‘driver’ option in the API server configuration file.

The API layer built on top of the database layer is responsible for all communications with the database layer. The API layer exposes the required functionality by means of HTTP endpoints. These endpoints act as a contract or interface to communicate with the service effectively preventing any direct access to the database. A formal specification of these endpoints is written in an API specification tool swagger (https://swagger.io/). It allows us to define all the aspects of the endpoint such as the name, input parameters and the format of the output. To ensure that the API is highly performant and reliable, we have built it in the Rust programming language (https://www.rust-lang.org) using the Actix web framework (https://actix.github.io).

The client layer is the public-facing interface of the iPTMnet service. It is the primary means by which users, as well as other software, can interact with the iPTMnet service. It consists of the iPTMnet website and the Python and R client packages.

### Client Packages

RESTful APIs, though relatively easy to use, require a certain level of technical expertise such as proficiency in the use of HTTP client libraries, as well as working knowledge of relevant HTTP codes and methods. These factors are a significant barrier for biologists. Considering that many biologists are proficient in the use of Python and R, we have created Python and R packages that cleanly wrap all the required code to communicate with the iPTMnet API. These packages provide functions that expose the required functionality in the form of well-defined functions. The client packages handle all the complexity of decoding the data received from the API and transforming it into the format requested by the user. This makes it easy to include the iPTMnet into other analysis pipeline.

The R package (iPTMnetR) is available through the CRAN repository and the Python package (PyiPTMnet) is available through the pip package manager. Installation instructions and a Quick Start guide for R package can be found at https://udel-cbcb.github.io/iPTMnetR/#/?id?iptmnetr and Python package can be found at https://udel-cbcb.github.io/pyiPTMnet/#/?id?pyiptmnet.

### Deployment

Deployment is the final piece in software development. Ensuring effortless and accurate deployment is crucial for the utility of the software. Deployment involves provisioning the production environment with the required operating system, packages, libraries and configuration files and brings all these components together to work as one unified system.

For iPTMnet, we have chosen to use Docker for deployment. Docker allows us to package all the required dependencies that include the userland of the preferred operating system, libraries and configuration files in clean redistributable Docker containers. These containers can then be executed by any user to reproduce the exact production environment on their own machines.

We provide three separate Docker containers for the website frontend, RESTful API and the database, respectively. This allows us to isolate the components from each other and choose the software stack that is appropriate for the specific component.

**Figure 2:**
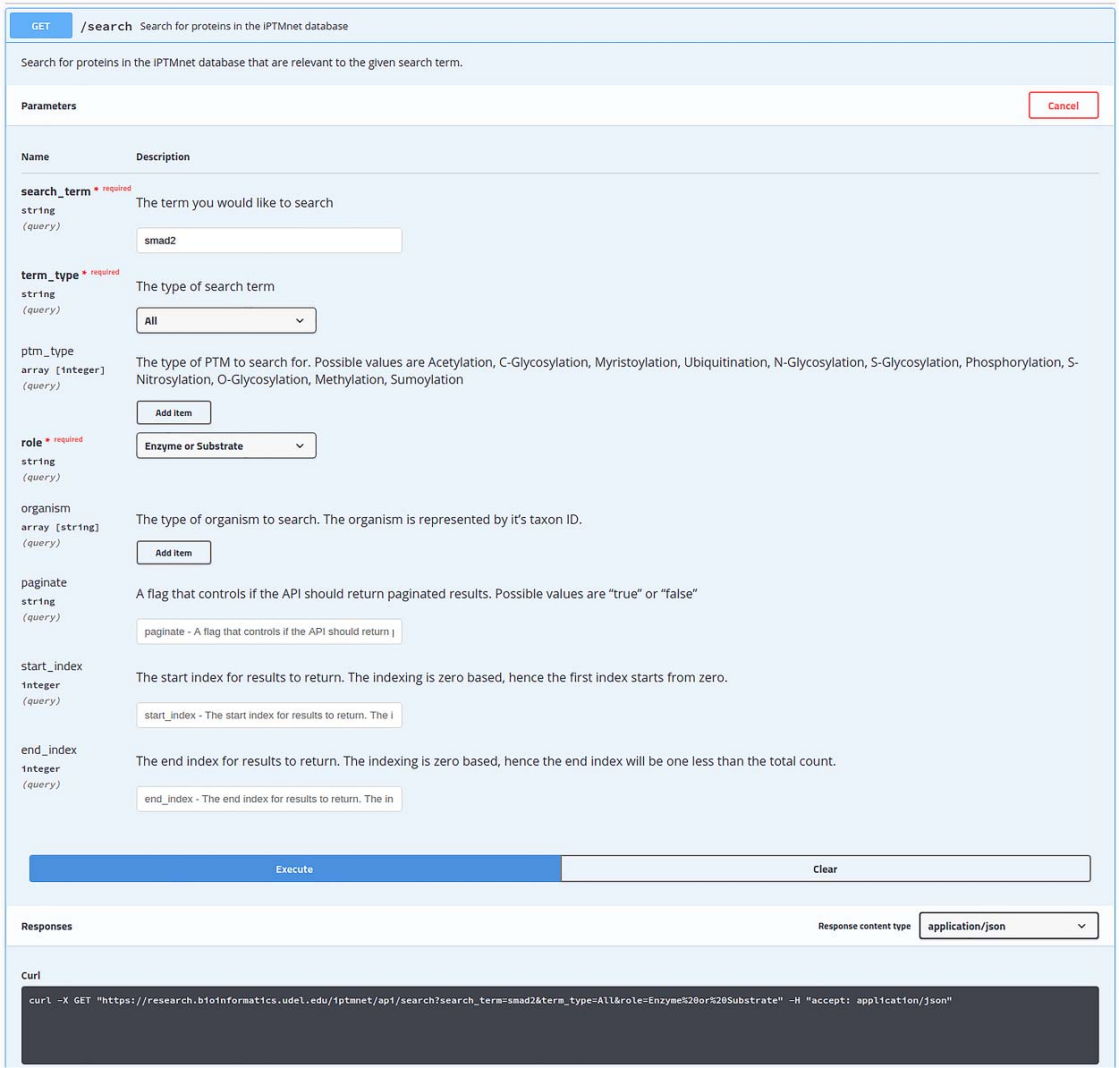
Performing a search with API using Swagger UI. iPTMnet API documentation for the ‘/search’ endpoint. The Swagger UI provides a webpage for users to explore the API in an interactive manner. Users can enter various query parameters and execute the query to obtain the results. The webpage also generates a cURL command (illustrated) that can be executed in a command-line shell to query the API and obtain the results.

**Figure 3:**
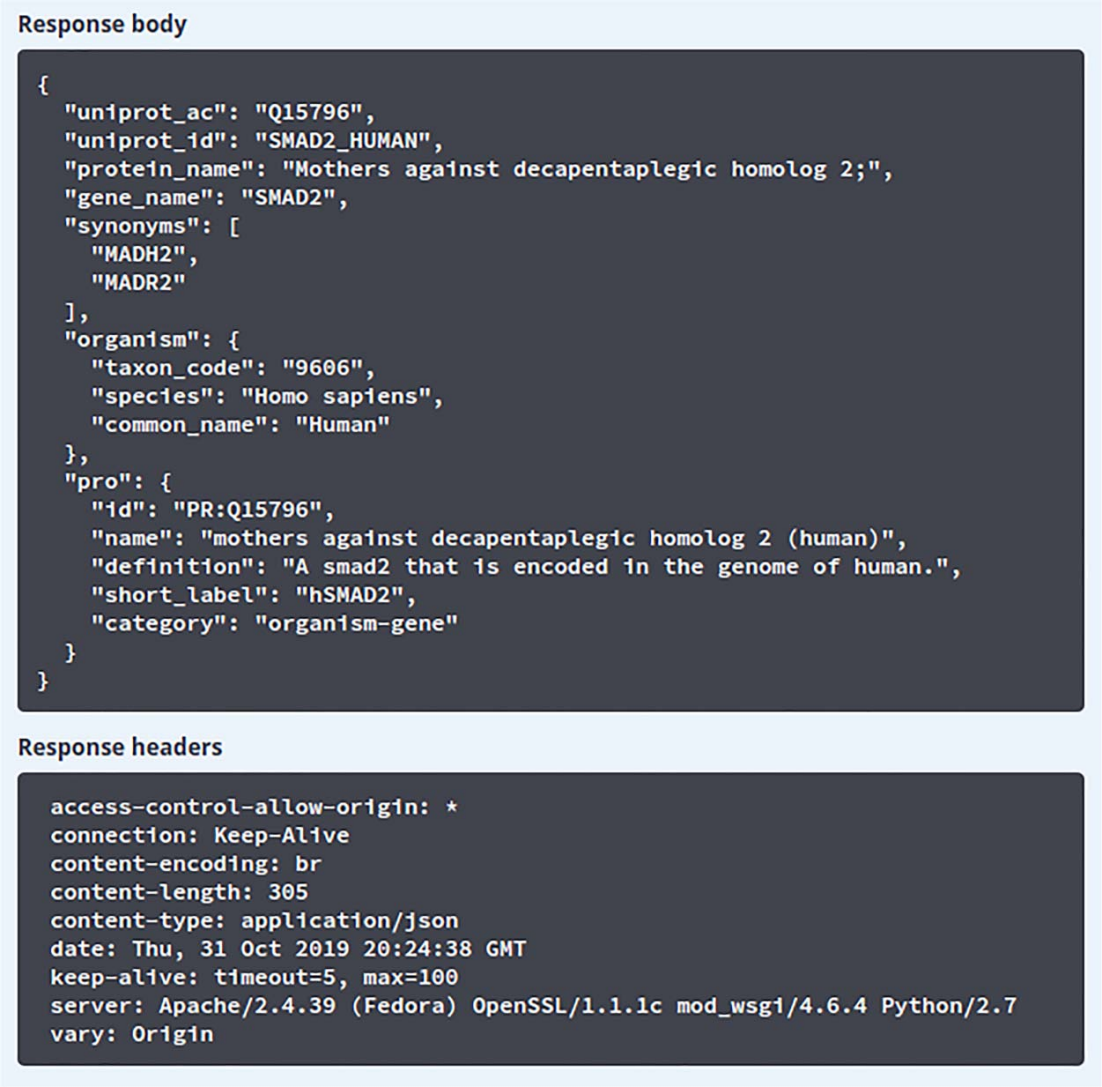
Result of search using Swagger UI. The results obtained after querying the ‘/info’ API endpoint. The response body section shows the JSON response received from the API server and the response headers section shows the HTTP headers from the received request.

The API documentation is provided using the swagger API documentation tool. The swagger UI allows users to perform test queries and explore the API. For example, as seen in Figure 2, the Swagger UI can be used to perform a search on iPTMnet API for the human (NCBI Taxonomic ID: code 9606) SMAD2 protein. Figure 3 shows the response returned by the server in JSON format along with response code and headers.

The API can also be accessed using the R and Python packages. For example, the following code snippet can be used to retrieve the list of PTM dependent PPIs for human SMAD2 (UniProt AC Q15796) (Table 2).

**Table 2:** Results obtained from the API

ptm_type	substrate_ uniprot_id	substrate_name	site	interactant_ uniprot_id	interactant_ name	association _type	source	pmid
Phosphorylation	P49841	GSK3B	S9	Q15796	SMAD2	Increased_ association	eFIP	21 996 745
Phosphorylation	Q15796	SMAD2	S465	Q13485	SMAD4	association	eFIP	9 346 908
Phosphorylation	Q15796	SMAD2	S467	Q13485	SMAD4	association	eFIP	9 346 908
Phosphorylation	Q15796	SMAD2	S465	Q13485	SMAD4	association	eFIP	9 346 966
Phosphorylation	Q15796	SMAD2	S467	Q13485	SMAD4	association	eFIP	9 346 966

**R:**

# load library.

library(iptmnetr).

# Get the ptm dependent ppi.

ptm_ppi < − get_ptm_dependent_ppi(“Q15796”).

**Python:**

# import the library.

import pyiptmnet.api as api.

# Get the ptm dependent ppi.

api.get_ptm_dependent_ppi(‘Q15796’).

In the next section, we will describe an extended scientific scenario that utilizes the R package to query iPTMnet.

#### Use case: connecting PTM sites to kinase signaling pathways

The development of mass spectrometry-based phosphoproteomics (20) has provided a great opportunity to explore global phosphorylation patterns in cells. While these studies produce a wealth of data on phosphorylation sites that are affected by disease or other perturbations, a necessary next step is to link these sites to their kinases and, ultimately, the upstream signaling pathways that are perturbing them. iPTMnet, a rich source of experimentally determined kinase–site relationships, can be a valuable resource for such analyses. Previously, we showed how to retrieve kinase information for sites of interest from a phosphoproteomic study manually using the batch retrieval tool on the iPTMnet website (1). With the iPTMnet API, the retrieval can be automated and incorporated into phosphoproteomic data analysis workflows.

In this section, we provide a tutorial in which we use the iPTMnet R package, iPTMnetR and iPTMnet v5.1 to re-analyze data from a published study (21) on the response of lung cancer cells to the tyrosine kinase inhibitor, erlotinib. Erlotinib is used as a therapeutic agent in lung cancer patients who carry mutations in the epidermal growth factor receptor (EGFR). Patients initially respond well to the drug but inevitably develop resistance. We focused on 243 phosphorylation sites in 194 proteins that were significantly upregulated by treatment with the EGFR ligand and “”epidermal growth factor (EGF) and downregulated by erlotinib (Supplementary Data 1). These sites are likely to be targets of EGFR-regulated pathways that are inhibited by drug treatment.

To retrieve kinase information from iPTMnet for the 243 EGFR/erlotinib-regulated sites, we used the function ‘get_ptm_enzymes_from_file’ (see Supplementary File 1 and Supplementary File 2, ‘Retrieving Kinase Information’ for the R and Python code, respectively). The only necessary parameter for the get_ptm_enzymes_from_file function is the name of the input file containing the site information (Supplementary Data 1.txt). This file should consist of three tab-delimited columns: UniProt AC of the phosphorylated protein, one letter code amino acid of the phosphorylated site and position of the phosphorylated site (e.g. P12345 S 100). The output, a data frame that is exported to a tab-delimited text file (Supplementary Data 2.txt), provides the UniProt AC and gene name of the PTM enzyme (e.g. kinase) and substrate, the modified site, the PTM type (e.g. phosphorylation), the confidence score on a scale of 0 (lowest confidence) to 4 (highest confidence) and the evidence source(s). Only sites that have at least one PTM enzyme are shown. If there are multiple kinases for a site, these are shown in multiple rows.

Using some simple R functions to manipulate the data frame containing the kinase information (see Supplementary File 1, ‘Basic Statistics’ for the R code used), we determined that there were a total of 118 kinase-site pairs involving 49 (20%) of the input sites and 53 kinases. The 53 kinases phosphorylated between one and six sites. Seventeen kinases phosphorylated three or more sites. The majority of the high-frequency retrieved kinases belong to signaling pathways that are known to be regulated by EGFR (22). From the PI3K/AKT/mTOR pathway, we identified AKT1 as well as three RPS6K family members, which are activated downstream of mTOR. From the PLC-gamma/DAG/PKC pathway, we identified three PKC family members. From the RAS/RAF/MEK/ERK pathway, we identified two MEKs (MAP2K1 and MAP2K2) and two ERKs (MAPK1 and MAPK3). Finally, we identified EGFR itself; EGFR phosphorylated four sites in our dataset. Although the fraction of sites that had kinase information was relatively low (20%), we were able to connect the phosphoproteomic data to major parts of the EGFR signaling landscape.

## Discussion

In this paper, we have discussed the rationale and the software development approach that we have followed to build the iPTMnet RESTful API. The Rust programming language has allowed the API server to be reliable, performant and easy-to-maintain. Docker has allowed us to integrate all the components of the API in one unified package, which can be distributed easily to install the API server on any hardware. Python and R packages have made the iPTMnet API accessible to users without the technical know-how to use the API directly. The API has also formalized the communications with the iPTMnet database allowing other tools to interact with iPTMnet without them being dependent on the underlying implementation. We further demonstrated how the IPTMnet API can be used to automate the data retrieval and subsequently integrate it into existing phosphoproteomic analysis workflows.

In conclusion, the RESTful API is a useful addition that benefits both the researchers and the developers of the iPTMnet service. The API also represents a FAIR model (23) for using the iPTMnet data.

## 

Project Name: iPTMnet RESTful API.

Project home page: https://research.bioinformatics.udel.edu/iptmnet/about/api

Operating System(s): Linux.

Programming language: Rust.

Other requirements: Glib 2.18 or higher, Docker 18.03.0 or higher.

License: MIT for software code and packages. CC BY-NC-SA 4.0 for the database.

Any restrictions to use by non-academics: CC BY-NC-SA 4.0.

## Supplementary Data

Availability of data and materials

Source code: https://github.com/udel-cbcb

Docker Repo: https://hub.docker.com/u/udelcbcb/

Data: https://doi.org/10.7910/DVN/UEIGXF

Client Packages

1. iPTMnetR: https://udel-cbcb.github.io/iPTMnetR/#/quickstart?id=info
2. PyiPTMnet: https://udel-cbcb.github.io/pyiPTMnet/#/quickstart?id=info

## Supplementary Material

Supplementary_Data_1

Supplementary_Data_2

Supplementary_File_1

Supplementary_File_2
