# Supplementary material for: RESTful API for iPTMnet: a resource for protein post-translational modification network discovery
Source: Database (Oxford). 2020 May 12;2020:baz157. doi: 10.1093/database/baz157 (PMC7216315; doi:10.1093/database/baz157)
Supplement: Supplementary_File_1 [file supplementary_file_1.pdf]

iptmnetr_use_case

Introduction

In this markdown, we re-analyze data from a published study on response of lung cancer cells to the tyrosine kinase inhibitor, erlotinib (PMID: 25404012). Erlotinib is used as a therapeutic agent in lung cancer patients who carry mutations in the epidermal growth factor receptor (EGFR). Patients initially respond well to the drug, but inevitably develop resistance. We focus on 243 phosphorylation sites in 194 proteins that were significantly upregulated by treatment with the EGFR ligand, epidermal growth factor (EGF), and downregulated by erlotinib. These sites are likely to be targets of EGFR-regulated pathways that are inhibited by drug treatment. We retrieve kinases for these sites from iPTMnet using iptmnetr and then compute some basic statistics on the results.

Retrieving Kinase Information

In this part, we retrieve kinases from iPTMnet for the EGFR/erlotinib-regulated sites using iptmnetr, and write the table of kinase-site relationships to a file. The sites are listed in the file egfr_sites_formatted.txt. The input file has three tab-delmited columns: UniProtAC of the phosphorylated protein, amino acid residue of the phosphorylated site, and position of the phosphorylated site (e.g., P12345 S 100).

```
#Set working directory
setwd("~/Documents/Bioinformatics/PIR/Publications/iPTMnet_api/iptmnet_api2")
#Load iptmnetr package
library("iptmnetr", lib.loc="/Library/Frameworks/R.framework/Versions/3.5/Resources/library")
kinase_info <- get_ptm_enzymes_from_file("Supplementary Data 1.txt")
write.table(kinase_info, file="Supplementary Data 2.txt", sep = '\t', quote = FALSE, col.names = NA)
head(kinase_info)
```

```
##   enz_name enz_id sub_name sub_id        ptm_type site site_position score
## 1     SGK1 O00141    NDRG1 Q92597 Phosphorylation S330           330     4
## 2    EEF2K O00418    EEF2K O00418 Phosphorylation T353           353     4
## 3    CHEK1 O14757   PRRC2B Q5JSZ5 Phosphorylation S166           166     1
## 4    CHEK1 O14757  PROSER2 Q86WR7 Phosphorylation  S43            43     1
## 5    CHEK1 O14757  RACGAP1 Q9H0H5 Phosphorylation S203           203     1
## 6    CHEK1 O14757    SENP2 Q9HC62 Phosphorylation S344           344     1
##                source                               pmids
## 1  neXtProt,PSP,Signor                   15461589,20416281
## 2 neXtProt,PSP,UniProt 22669845,22329831,22216903,23186163
## 3                  PSP                            21851590
## 4                  PSP                            21851590
## 5                  PSP                            21851590
## 6                  PSP                            21851590
```

Basic Statistics

Next, we compute:

• Number of kinase-site pairs

• Number of sites with at least one kinase

• Number of kinases

• Number of sites per kinase

• Number of kinases that phosphorylate three or more sites

```
#Find number of kinase-site pairs
num_kinase_site_pairs <- nrow(kinase_info)
num_kinase_site_pairs
```

```
## [1] 118
```

```
#Find number of sites with at least one kinase
kinase_info$full_site <- paste(kinase_info$sub_id, kinase_info$site, sep = " ")
dup_sites <- duplicated(kinase_info$full_site)
num_unique_sites <- nrow(kinase_info[!dup_sites,])
num_unique_sites
```

```
## [1] 49
```

```
#Find number of unique kinases
num_unique_kinases <- nrow(kinase_info[!duplicated(kinase_info$enz_id),])
num_unique_kinases
```

```
## [1] 53
```

```
#Find number of sites per kinase (uses the plyr package)
library("plyr", lib.loc="/Library/Frameworks/R.framework/Versions/3.5/Resources/library")
kinase_tally <- count(kinase_info, "enz_name")
kinase_tally_sorted <- kinase_tally[order(-kinase_tally$freq),]
kinase_tally_sorted
```

```
##    enz_name freq
## 35    PRKCA    6
## 50  RPS6KB1    6
## 20    MAPK1    5
## 36    PRKCD    5
## 46  RPS6KA1    5
## 2      AKT1    4
## 8     CHEK1    4
## 11     EGFR    4
## 18   MAP2K2    4
## 34   PRKACA    4
## 5     AURKB    3
## 17   MAP2K1    3
## 23    MAPK3    3
## 33     PLK1    3
## 37    PRKCE    3
## 40    PRKD1    3
## 47  RPS6KA3    3
## 6      BRAF    2
## 7    CAMK2A    2
## 9   CSNK2A1    2
## 13    IKBKB    2
## 15     JAK2    2
## 19   MAP3K8    2
## 29     PAK2    2
## 31    PDPK1    2
## 38    PRKCH    2
## 39    PRKCZ    2
## 41    PRKD3    2
## 44    ROCK1    2
## 49  RPS6KA5    2
## 52      SRC    2
## 1      ABL1    1
## 3      AKT2    1
## 4     AURKA    1
## 10    EEF2K    1
## 12      HCK    1
## 14     INSR    1
## 16      LCK    1
## 21   MAPK13    1
## 22   MAPK14    1
## 24    MAPK8    1
## 25 MAPKAPK5    1
## 26    MKNK1    1
## 27     MTOR    1
## 28     PAK1    1
## 30     PASK    1
## 32     PKD1    1
## 42     PTK6    1
## 43      RET    1
## 45    ROCK2    1
## 48  RPS6KA4    1
## 51     SGK1    1
## 53      US3    1
```

```
#Find number of kinases that phosphorylate three or more sites
high_freq_kinases <- kinase_tally_sorted[ which(kinase_tally_sorted$freq >= 3),]
num_high_freq_kinases <- nrow(high_freq_kinases)
num_high_freq_kinases
```

```
## [1] 17
```
