# Supplementary material for: RESTful API for iPTMnet: a resource for protein post-translational modification network discovery
Source: Database (Oxford). 2020 May 12;2020:baz157. doi: 10.1093/database/baz157 (PMC7216315; doi:10.1093/database/baz157)
Supplement: Supplementary_File_2 [file supplementary_file_2.pdf]

iptmnet_use_case

December 6, 2019

0.1 Introduction

In this markdown, we re-analyze data from a published study on response of lung cancer cells to the tyrosine kinase inhibitor, erlotinib (PMID: 25404012). Erlotinib is used as a therapeutic agent in lung cancer patients who carry mutations in the epidermal growth factor receptor (EGFR). Patients initially respond well to the drug, but inevitably develop resistance. We focus on 243 phosphorylation sites in 194 proteins that were significantly upregulated by treatment with the EGFR ligand, epidermal growth factor (EGF), and downregulated by erlotinib. These sites are likely to be targets of EGFR-regulated pathways that are inhibited by drug treatment. We retrieve kinases for these sites from iPTMnet using iptmnetr and then compute some basic statistics on the results.

0.2 Retrieving Kinase Information

In this part, we retrieve kinases from iPTMnet for the EGFR/erlotinib-regulated sites using pyiptmnet, and write the table of kinase-site relationships to a file. The sites are listed in the file egfr_sites_formatted.txt. The input file has three tab-delmited columns: UniProtAC of the phosphorylated protein, amino acid residue of the phosphorylated site, and position of the phosphorylated site (e.g., P12345 S 100).

[1]:
```
import pyiptmnet.api as api
```

[2]:
```
kinase_info =  api.get_ptm_enzymes_from_file("Supplementary Data 1.txt")
kinase_info = kinase_info.sort_values(by="enz_name")
kinase_info.head()
```

[2]:
```
   enz_name  enz_id sub_name  sub_id         ptm_type   site  site_position  \
20     ABL1  P00519     EGFR  P00533  Phosphorylation  Y1197           1197
63     AKT1  P31749    PDCD4  Q53EL6  Phosphorylation   S457            457
62     AKT1  P31749     FLNC  Q14315  Phosphorylation  S2233           2233
60     AKT1  P31749    EIF4B  P23588  Phosphorylation   S422            422
61     AKT1  P31749     IRS1  P35568  Phosphorylation   S629            629

    score               source     pmids
20      3  neXtProt,PSP,Signor  16943190
63      3  neXtProt,PSP,Signor  16357133
62      3         neXtProt,PSP  15461588
```

```
60      3      neXtProt,Signor  18836482
61      3         neXtProt,PSP  17640984
```

[3]:
```
kinase_info.to_csv("Supplementary Data 2.txt",sep='\t')
```

0.3 Basic Statistics

Next, we compute:

- Number of kinase-site pairs
- Number of sites with at least one kinase
- Number of kinases
- Number of sites per kinase
- Number of kinases that phosphorylate three or more sites

[5]:
```
#Find number of kinase-site pairs
num_kinase_site_pairs = len(kinase_info)
display(num_kinase_site_pairs)
```

```
118
```

[7]:
```
#Find number of sites with at least one kinase
kinase_info["full_site"] = kinase_info["sub_id"] + " " + kinase_info["site"]
unique_sites = kinase_info.drop_duplicates(subset="full_site")
len(unique_sites)
```

[7]: 49

[8]:
```
#Find number of unique kinases
unique_kinases = kinase_info.drop_duplicates(subset="enz_id")
len(unique_kinases)
```

[8]: 53

[9]:
```
#Find number of sites per kinase
kinase_tally_sorted  = kinase_info["enz_name"].value_counts()
kinase_tally_sorted
```

[9]:
```
PRKCA       6
RPS6KB1     6
RPS6KA1     5
MAPK1       5
PRKCD       5
AKT1        4
EGFR        4
MAP2K2      4
```

```
CHEK1       4
PRKACA      4
MAP2K1      3
AURKB       3
MAPK3       3
RPS6KA3     3
PRKCE       3
PLK1        3
PRKD1       3
PRKD3       2
PRKCZ       2
BRAF        2
PAK2        2
MAP3K8      2
RPS6KA5     2
CSNK2A1     2
PRKCH       2
JAK2        2
ROCK1       2
SRC         2
IKBKB       2
CAMK2A      2
PDPK1       2
MAPK14      1
MAPK13      1
AKT2        1
ABL1        1
MTOR        1
MAPK8       1
RET         1
ROCK2       1
RPS6KA4     1
AURKA       1
US3         1
PKD1        1
PASK        1
MKNK1       1
EEF2K       1
LCK         1
MAPKAPK5    1
PAK1        1
INSR        1
SGK1        1
HCK         1
PTK6        1
Name: enz_name, dtype: int64
```

[10]:
```python
#Find number of kinases that phosphorylate three or more sites
high_freq_kinases = kinase_tally_sorted[kinase_tally_sorted >= 3]
num_high_freq_kinases = len(high_freq_kinases)
num_high_freq_kinases
```

[10]: 17
